# Case Report: Two cases of rare head injuries from Nepal

**DOI:** 10.12688/f1000research.16225.1

**Published:** 2018-09-18

**Authors:** Joe M. Das, Apar Pokharel, Rashmi Sapkota, Manish Mishra, Ashish Babu Aryal

**Affiliations:** 1 Department of Neurosurgery, College of Medical Sciences - Teaching Hospital, Bharatpur - 10, Chitwan, 44200, Nepal; 2 Department of Otorhinolaryngology, College of Medical Sciences - Teaching Hospital, Bharatpur - 10, Chitwan, 44200, Nepal

**Keywords:** Craniocerebral trauma, Craniotomy, Skull base, Frontal sinus, Cerebrospinal fluid, Contusions

## Abstract

**Background:** There are a number of ways in which one can sustain a head injury. Even if you are doing simple household activities or going out for a morning walk, you cannot be sure of what type of injury awaits you. The source of injury may be a pressure cooker whistle acting as a projectile or a hailstone falling from the sky. Such injuries are common in Nepal, considering the socio-demographic and geographic conditions. In this article, we present two such very rare cases of head injury.

**Case Reports:** The first case is a middle-aged woman who sustained an accidental injury to the face associated with fracture of frontal sinus and frontal contusion, following the impact from a high momentum projectile in the form of the pressure regulator of a pressure cooker. She underwent craniotomy and removal of the foreign body. In the second case, an elderly man sustained minor injury to the head following the fall of hail. The abrasions and contusions produced by the hail were managed conservatively. Since he did not have any clinical evidence of head injury, other than multiple abrasions with contusions in the scalp, he did not undergo any imaging studies. He did not have any neurological deficits. The postoperative period was uneventful for the first patient and she was followed up for one month. The second patient was lost to follow-up.

**Conclusion:** Successful management of two very rare cases of head injuries from Nepal are reported. Proper care and maintenance of the house-hold utensils that are constantly used may protect people from head injuries. Though natural calamities cannot always be avoided, simple measures like using an umbrella while going outdoors may protect individuals from head injuries due to hailstones.

## Introduction

The pressure cooker (PC) is an essential utensil for cooking in Asian kitchens, especially in Nepal and India, and are mostly handled by women. Accidental injury from such a commonly used utensil can sometimes be grievous. Most of the reports of injuries from PCs concern burns due to sudden opening of the lid and releasing of steam under very high pressure ^1^. There is one previous report in which a mandible fracture occurred along with burns ^2^ and one in which brain damage occurred due to the cooker blast ^3^. However, there are only five reports of accidental head injury due to a pressure regulator projectile to date ^4– 8^. Ours is the sixth case, and the second producing a craniofacial injury, published in the literature, which describes successful management.

Hail is a variant of weather which can be occasionally harmful as well as dangerous. It is a form of precipitation and consists of balls or irregular lumps of ice known as hailstones. Hailstones are composed mostly of ice and measure 5–50 mm in diameter. Hail is produced by cumulonimbus clouds otherwise known as thunderstorm clouds, which are transparent ice or made up of alternating layers of transparent and translucent ice, at least 1 mm thick ^9^. Though hailstones are of small size usually, sometimes they are large enough to kill a person if it falls on the head. A previous newspaper report of a “hailstone massacre”, which occurred centuries ago details this scenario ^10^. The second case reported here is a case of mild head injury produced by falling hailstones. Surprisingly, head injury produced by hailstone has never been reported previously in the literature, to the best of our knowledge.

## Case 1

A 55-year-old woman, with no known comorbidities, was cooking dal using a pressure cooker at night. The patient doubted why there was no whistling after the expected time, and so tried to gently lift the pressure regulator, which suddenly gave way and was thrust into her face, like a projectile, near the right eye. There was no history of loss of consciousness, nasal bleed or seizures, though she had one episode of vomiting. She was a chronic smoker and occasional drinker of alcohol.

**Figure 1.**
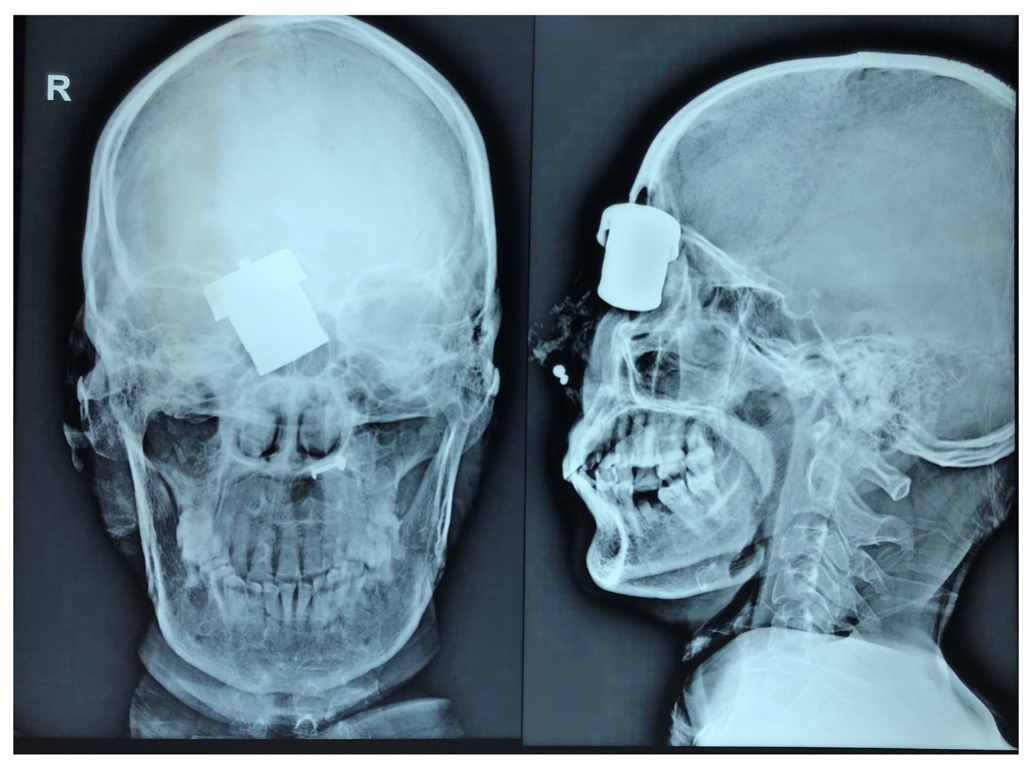
Plain X-ray of skull, antero-posterior and lateral views showing the radio-opaque foreign body stuck in the anterior cranial fossa floor.

**Figure 2.**
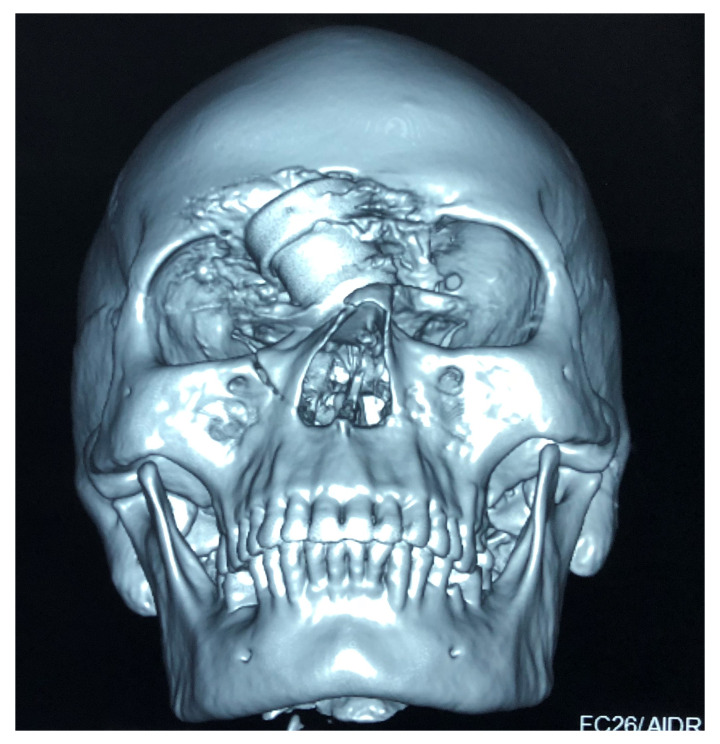
Plain Computed Tomogram of the face and skull (3D reconstruction) showing the foreign body and its relation to the right orbit.

**Figure 3.**
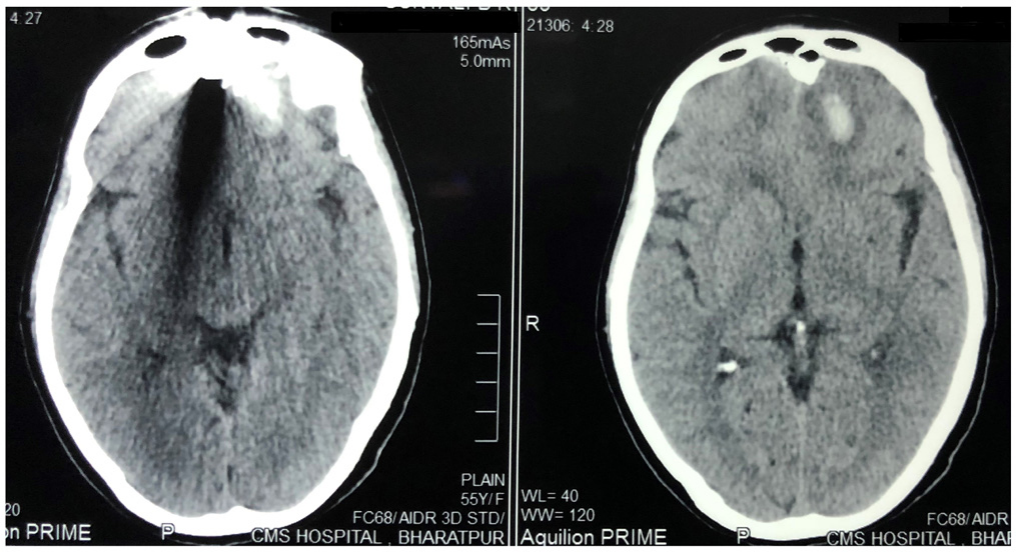
Plain Computed Tomogram of the brain showing the left frontal contusion and metal artefact due to foreign body.

When she reached our emergency room in June 2018, her vital signs were stable. She had a wound of size 4×2 cm in between the right eye and root of nose, which was deep and still had the cooker whistle lodged. There was cerebrospinal fluid (CSF) mixed with blood in the periphery of the wound and there was mechanical ptosis of right eye. The patient was conscious and oriented. Emergency X-ray of skull ( Figure 1) and computed tomography (CT) of the head ( Figure 2) were done, which showed the foreign body just lateral to the root of nose on the right side with the right lateral wall of the nostril fractured and pushed inside. There was fracture involving the right frontal sinus with pneumocephalus and 1×1 cm sized left frontal contusion ( Figure 3).

**Figure 4.**
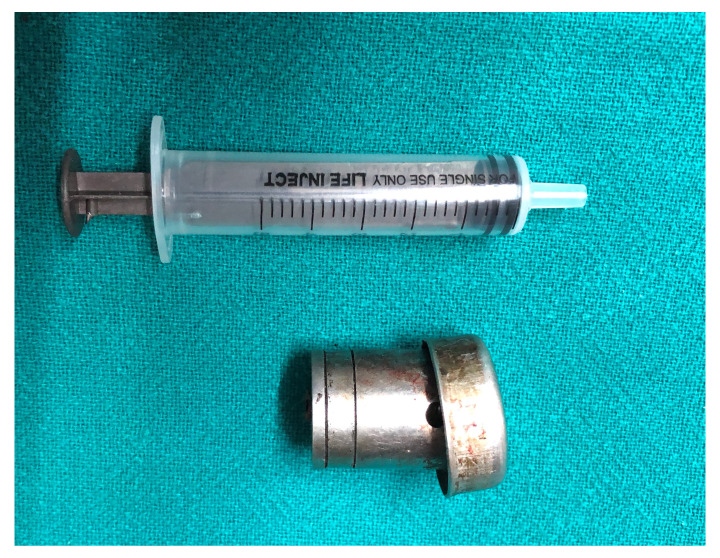
The retrieved foreign body measuring 3.0 × 2.5 cm in size.

**Figure 5.**
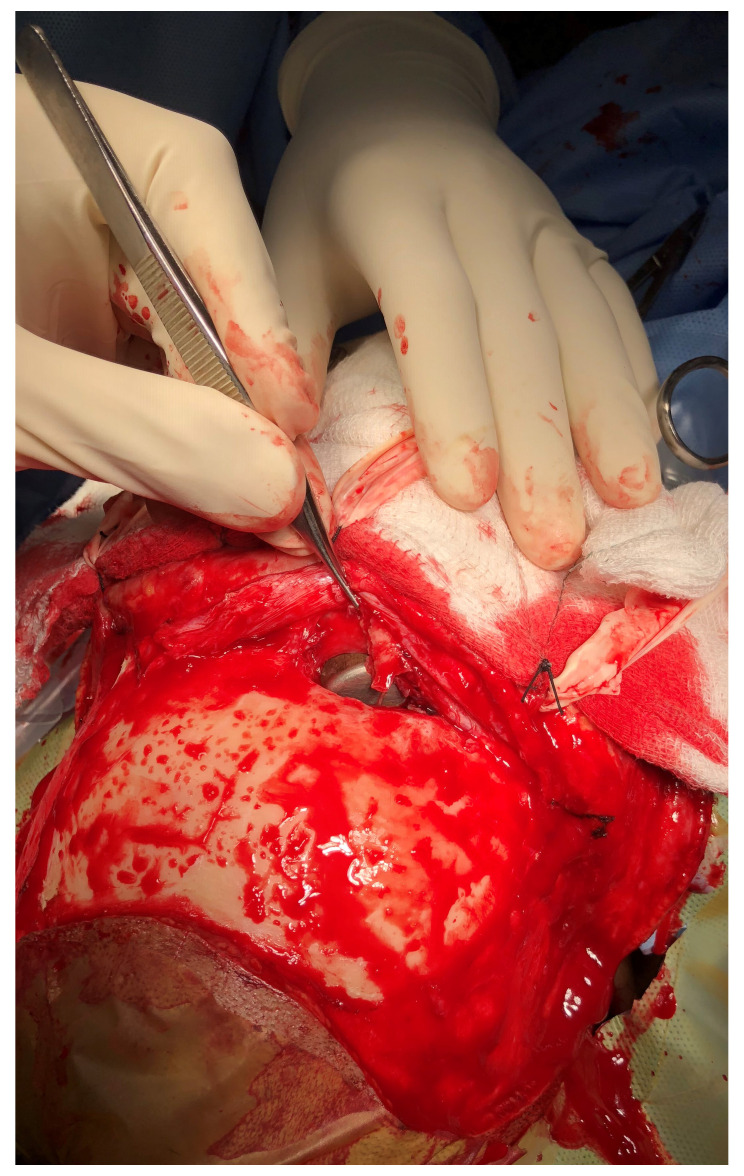
Intra-operative picture showing the fractured right frontal sinus with the foreign body *in situ*.

The patient underwent emergency bifrontal craniotomy. The right frontal sinus fracture with dural tear of size 0.5×1 cm was noted and the foreign body ( Figure 4) was retrieved through the fracture from the cranial aspect ( Figure 5). The frontal sinus was exteriorized and packed with muscle and bone wax applied across the defect. Anterior cranial fossa was carpeted with pedicled pericranial flap and the wound was closed after replacing the bone flap. The wound at the site of foreign body was seen by our otorhinolaryngologist and was closed in layers. The nasal mucosa was intact and there was no injury to nasolacrimal duct. The fractured nasal bone was not disturbed as it could cause stenosis of the right nostril.

The patient was started on Meropenem 2 g thrice a day, Vancomycin 1 g twice a day and Metronidazole 500 mg thrice a day as antibiotics, and levetiracetam 500 mg twice a day as anticonvulstant, which were continued for one week. The patient’s vision was fully preserved. The post-operative period was uneventful. Post-operative CT scan of brain showed resolving frontal contusion and she was discharged on post-operative day 8. When she came for follow-up after one month, she was asymptomatic, the wound had healed fully and there was no CSF leak.

## Case 2

An 85-year-old man presented to our neurosurgery out-patient department in May 2018 with complaint of headache following falling of hailstones on his head three days previously. He was a chronic smoker and alcoholic and did not have any comorbidities. He had no history of previous hospital admissions.

**Figure 6.**
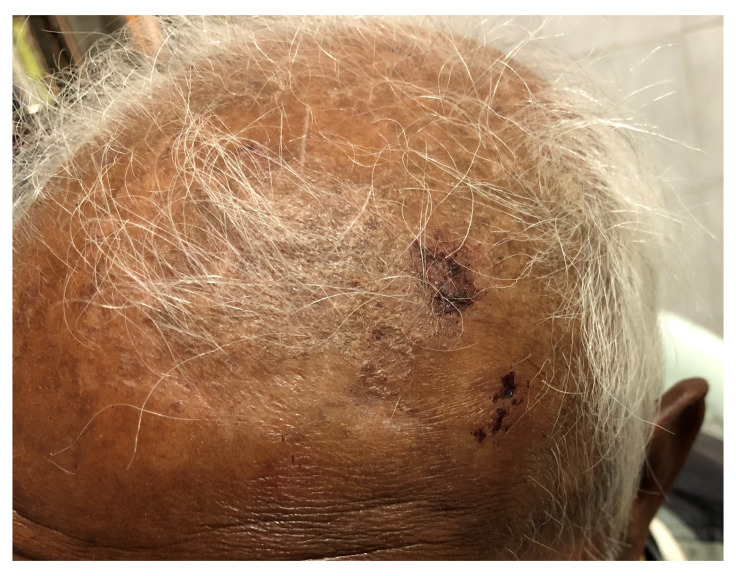
Scalp contusions and abrasions in the left frontal region produced by the impact of falling hailstones.

Following the incident, the patient did not lose consciousness, vomit, or have a seizure. He also did not have any nasal or ear bleed. On clinical examination, he was neurologically intact and there was no papilledema on examination of the fundus. The patient had three healing abrasions with contusions, each the size of around 1 × 1 cm with local tenderness. Two abrasions were located in the left frontal region, behind hairline and one in the right parietal region, behind hairline ( Figure 6). There was no clinical evidence of infection or skull fracture. Radiological imaging was not warranted and he was managed with 37.5 mg of tramadol hydrochloride and 325 mg of acetaminophen twice a day for five days. Though he was instructed to come for follow-up after one week, he did not turn up.

## Discussion

The pressure cooker (PC), invented by the French-born British physicist Denis Papin in 1679, is a hermetically sealed pot that produces steam heat to cook food quickly. The PC heats water to produce very hot steam and as a result, the temperature inside it will increase to around 130° C, which is much higher than the maximum heat produced by ordinary cookware. The main advantage of this much high temperature is that it penetrates food quickly so that cooking time is reduced without diminishing vitamin and mineral content ^11^. The problem faced high altitude areas, like Nepal, is that boiling happens at low temperature due to reduced atmospheric pressure. PCs will increase the pressure so that cooking occurs at the appropriate temperature.

PC pressure regulator projectile injury to the face was first reported by Chattopadhyay *et al* in 2010 ^4^. Altogether, there are five reports of this type of case ( Table 1), four of them with significant ocular trauma. The case reported by Gupta *et al.* was similar to our case and had significant head injury ^5^. In that case, the foreign body lodged transorbitally and was operated on promptly, even though the patient’s vision was lost by the injury and led to the development of a brain abscess subsequently. Our patient was lucky enough for her eye to escape from the direct impact of foreign body and from further complications of surgery.

**Table 1.** Reports of craniofacial injuries caused by pressure regulator projectile of pressure cookers to date.

Author (year)	Age of patient	Sex	Site of impact	Neurological examination	X-ray/CT finding	Structures involved	Procedure done	Outcome
Chattopadhyay SS *et al*. (2010) ^4^	32 y	F	Right upper eye lid	Vision – PL GCS-13	Disorganized globe with impacted nozzle	Right globe with autoevisceration of ocular contents	Glass ball ocular implant was placed and scleral laceration repaired	Not mentioned
Gupta OP *et al*. (2013) ^5^	47 y	F	Left forehead and eye	Vision – No PL GCS – 13	Metallic foreign body inside left orbit with basifrontal contusion with pneumocephalus	Left orbital wall, frontal sinus and globe	Evisceration of left eye with removal of foreign body	Developed brain abscess after 1 month, which was evacuated. Final neurological outcome - good
Dobariya *et al*. (2014) ^6^	29y	M	Left upper eye lid	Vision – No PL GCS – 14	Intruded whistle in left orbit. No fracture	Left globe with autoevisceration of ocular contents	Scleral laceration repaired	Not mentioned
Atreya *et al*. (2016) ^7^	62y	F	Right parotid region	Right LMN facial palsy	Rectangular shadow over mandibular region	Subcutaneous tissue and facial nerve	Removal and primary closure	Not mentioned
Singh AK *et al*. (2016) ^8^	26 y	F	Between root of nose and right eye ball	Vision – No PL GCS – 15	Fracture of medial orbital wall with penetration of the foreign body for 3 centimetres inside the bony orbit	Right globe with autoevisceration and exposed uveal tissue with oedematous upper lid	Evisceration, suturing of sclera and conjunctiva. Skin lacerations left to heal by secondary intention	3 months - referred to ocularist for custom- made ocular prosthesis
Our case	55 y	F	Between root of nose and right eye ball	Vision – Normal GCS – 15	Foreign body just lateral to the root of nose on the right side with the right lateral wall of nostril fractured and pushed inside. There was fracture involving the right frontal sinus with pneumocephalus and 1×1 cm sized left frontal contusion	Right orbital wall, nasal bone, frontal sinus, frontal lobes	Bifrontal craniotomy and removal of foreign body followed by primary closure	1 month – Neurologically intact

CT – Computed tomogram, y – years, M – Male, F – Female, PL – Perception of light, GCS – Glasgow Coma Scale Score, LMN – Lower motor neuron

PCs can maintain high temperature (121°C) and proper pressure (1 kg/cm ^2^) inside for cooking. The pressure is controlled inside by the vent weight (pressure regulator) and its spring action - excess steam goes out through the vent tube. Sometimes it can get blocked due to imperfect cleaning, excessive volume of water or overfilling with green leaves ^12^. Then excess steam will accumulate inside, which can push the PC’s lid or pressure regulator out with huge force. Such accidents can be reduced by proper maintenance of the cooker, cleaning the lid and vent valve and filling the objects inside the cooker up to the appropriate level.

The projectile of a pressure regulator will almost always be directed towards the face (especially orbit) and skull. As has been reported previously, it is always safe to perform a craniotomy followed by removal of the foreign body for all foreign bodies, which have breached the dura, to prevent inadvertent damage to vital structures ^13^. The patient had pneumocephalus with frontal sinus fracture, which was suggestive of a breach in the dura. If the foreign body is directly taken out blindly without exposing it through craniotomy, there is a chance that injury can occur to the brain as well as to the bridging vein at anterior skull base, if any ^14^. This was the rationale for approaching the foreign body via cranium.

Hail is a form of frozen precipitation (hydrometeor) which originates in a thunderstorm cloud, scientifically known as cumulonimbus (thundercloud), which is composed of water droplets and ice crystals. There are upward forces in such clouds known as updrafts, and they carry raindrops upward into very cold areas of the atmosphere. In such areas, water droplets become super-cooled and freeze when coming into contact with condensation nuclei (small aerosols), thus forming small hailstones. The updraft then dissipates and these hailstones fall down. But these will be brought back into another updraft, and will be lifted up again. A layer of ice will get added to the hailstone and it grows in size with each ascent. Once a hailstone becomes too heavy to be supported by the updraft, it falls down from the cloud ^15^. The main factors present in thunderstoms that are favorable to hail formation are strong updrafts, large liquid water contents, large cloud-drop sizes, and great vertical height ^16^. Hail usually falls during severe thunderstorms in the warm season, when the temperature on the surface of the earth rises above 20 °C ^9^.

Hailstone is an individual unit of hail. By convention, any frozen precipitation having a diameter of 5 mm or more is classified as hailstone, whereas smaller particles of similar origin are known as either ice pellets or snow pellets ^17^. In the Cambridge dictionary, hailstone is defined as “a small, hard ball of ice that falls from the sky like rain” ^18^. Most of the hail storms are made up of hailstones of different sizes. Usually only the large ones pose serious risk to people caught in the open. According to the Guinness book of world records, the heaviest hailstones ever recorded weighed approximately 1 kilogram and are reported to have killed 92 people in the Gopalganj area of Bangladesh on 14 April 1986 ^19^. The largest hailstone recently recovered in the USA fell in Vivian, South Dakota on June 23, 2010 with a diameter of 8 inches and a circumference of 18.62 inches. It also weighed almost 1 kilogram ^20^. One of the most lethal hailstorms in history, leading to the death of hundreds of nomads, occurred around AD 850 close to the glacial Roopkund Lake in the remote Himalayan Gahrwal region ^10^.

Even though there is a high frequency of occurrence of thunderstoms in the tropics, hail is actually less common in these regions, compared to the mid-latitudes, as the atmosphere over the tropics is warmer over a much greater height. Hail is common in mountain ranges because mountains force horizontal winds to move suddenly upwards (orographic lifting). This intensifies the updrafts within thunderstorms which makes hail more likely ^21^. Hence hail is relatively common in Nepal.

There has been no report of hail falling on the head and producing injury, though hail is known to cause widespread damage to farms, houses, animals and humans. The present case did not have any criteria for radiological imaging as per the Canadian CT rule ^22^. Moreover, the patient presented three days after the incident. In Nepal, patients may not be presenting immediately after the injury, either because they have only mild symptoms or they have to travel a long distance to reach a tertiary care hospital. The main limitation of this case is that there is no proper follow-up.

## Conclusion

Head injury can occur wherever you are – it does not matter whether you are indoors or outdoors. Here this fact is stressed with the help of two different and extremely rare types of head injuries. Some simple manoeuvres, such as proper maintenance of equipment and utensils you are working with or taking an umbrella while going for a morning walk, may protect individuals from such calamities.

## Consent

Written informed consent for publication of their clinical details and/or clinical images was obtained from both patients.

## Data availability

All data underlying the results are available as part of the article and no additional source data are required.
