# Supplementary material for: Case Report: Two cases of rare head injuries from Nepal
Source: F1000Res. 2021 Feb 22;7:1483. Originally published 2018 Sep 18. [Version 2] doi: 10.12688/f1000research.16225.2 (PMC11541069; doi:10.12688/f1000research.16225.2)
Supplement: Peer review history

# Reviewer report: Reviewer response for version 2

**DOI:** 10.5256/f1000research.30538.r320004

**Published:** 2024-11-06

**Authors:** Mustafa Ismail

**Affiliations:** 1 University of Baghdad, Baghdad, Iraq

This article provides valuable insights into rare head injuries from unconventional causes, such as a pressure cooker projectile and a hailstorm. Its detailed case reports highlight the importance of proper safety measures and awareness in preventing such incidents. The successful management of these injuries enhances clinical understanding and serves as a useful reference for neurosurgeons encountering similar cases.

Are enough details provided of any physical examination and diagnostic tests, treatment given and outcomes?

Yes

Is the case presented with sufficient detail to be useful for other practitioners?

Yes

Is sufficient discussion included of the importance of the findings and their relevance to future understanding of disease processes, diagnosis or treatment?

Partly

Is the background of the case’s history and progression described in sufficient detail?

Yes

Reviewer Expertise:

Neurosurgery

I confirm that I have read this submission and believe that I have an appropriate level of expertise to confirm that it is of an acceptable scientific standard.

# Reviewer report: Reviewer response for version 2

**DOI:** 10.5256/f1000research.30538.r79981

**Published:** 2021-02-23

**Authors:** Amit Agrawal

**Affiliations:** 1 Department of Neurosurgery, Narayana Medical College and Hospital, Nellore, Andhra Pradesh, India

No new comments.

Are enough details provided of any physical examination and diagnostic tests, treatment given and outcomes?

Partly

Is the case presented with sufficient detail to be useful for other practitioners?

Partly

Is sufficient discussion included of the importance of the findings and their relevance to future understanding of disease processes, diagnosis or treatment?

Partly

Is the background of the case’s history and progression described in sufficient detail?

Partly

Reviewer Expertise:

NA

I confirm that I have read this submission and believe that I have an appropriate level of expertise to confirm that it is of an acceptable scientific standard.

# Reviewer report: Reviewer response for version 1

**DOI:** 10.5256/f1000research.17719.r67439

**Published:** 2020-08-12

**Authors:** Amos O Adeleye

**Affiliations:** 1 Department of Neurological Surgery, University College Hospital, Ibadan, Nigeria

This is generally an interesting clinical vignette by these authors, especially the first case. We are of the opinion actually that the authors have enough literature base as well as the clinical materials of the first case to do this write-up simply as a case-based literature review of head injury/traumatic brain injury from projectiles from house-hold pressure cookers.

 The second case does not warrant any scientific report in our opinion, not by the near normal clinical acute-event / immediate post-event period status of the patient, as well as the very minimal body harm to the patient.

 Having said that, we like to make some of the observations below concerning the authors’ otherwise well-written paper.

 The figure 2, the plain 3-D reconstruction of the cranial CT bone windows, contains more information that just ‘the foreign body and its relation to the orbit’. There is the impression of a right naso-orbital fracture extending from the inferomedial orbital rim across the maxilla (just medial to the zygomasticofaical foramen) to the right nasal wall. One would expect all this to reflect in the authors’ case description (and the figure legend) by way of emphasis on the apparent destructive impact of the house-hold utensil turned projectile.

 Case 1

 “A 55-year-old woman, with no known comorbidities, was cooking dal using a pressure….”

 What’s ‘dal’ please, for us international readers?

 “There was fracture involving the right frontal sinus with pneumocephalus and 1×1 cm sized left frontal contusion (Figure 3).”

 The figure 3 referred to here is a brain, not a bone, window of the cranial CT. The frontal sinus fracture being described could therefore hardly be appreciated. We had actually wondered why the authors did not render some of the images at this frontobasal level in the bone windows. That’s what would have been more instructive for this part of the work

Are enough details provided of any physical examination and diagnostic tests, treatment given and outcomes?

Partly

Is the case presented with sufficient detail to be useful for other practitioners?

Yes

Is sufficient discussion included of the importance of the findings and their relevance to future understanding of disease processes, diagnosis or treatment?

Yes

Is the background of the case’s history and progression described in sufficient detail?

Yes

Reviewer Expertise:

Clinical neurological surgery,

I confirm that I have read this submission and believe that I have an appropriate level of expertise to confirm that it is of an acceptable scientific standard, however I have significant reservations, as outlined above.

# Response

**Published:** 2020-10-30

**Authors:** Joe M Das

**Affiliations:** College of Medical Sciences - Teaching Hospital, Nepal

Thank you for looking into our article in detail and providing constructive feedback.

 The article has been amended as suggested.

1. The term "dal" has been explained.
2. A detailed description of the facial fracture is included.
3. A new figure showing the fractured frontal sinus has been provided.

# Response

**Published:** 2020-11-11

**Authors:** Joe M Das

**Affiliations:** College of Medical Sciences - Teaching Hospital, Nepal

This is generally an interesting clinical vignette by these authors, especially the first case. We are of the opinion actually that the authors have enough literature base as well as the clinical materials of the first case to do this write-up simply as a case-based literature review of head injury/traumatic brain injury from projectiles from house-hold pressure cookers. - **Thank you.**

 The second case does not warrant any scientific report in our opinion, not by the near normal clinical acute-event / immediate post-event period status of the patient, as well as the very minimal body harm to the patient. - **We still would like it to be published as there are not many reports on the harms this natural calamity can do to humans.**

 Having said that, we like to make some of the observations below concerning the authors’ otherwise well-written paper.

 The figure 2, the plain 3-D reconstruction of the cranial CT bone windows, contains more information that just ‘the foreign body and its relation to the orbit’. There is the impression of a right naso-orbital fracture extending from the inferomedial orbital rim across the maxilla (just medial to the zygomasticofaical foramen) to the right nasal wall. One would expect all this to reflect in the authors’ case description (and the figure legend) by way of emphasis on the apparent destructive impact of the house-hold utensil turned projectile. - **We have added the details of the fracture in the case description and figure legend, as suggested**.

 Case 1

 “A 55-year-old woman, with no known comorbidities, was cooking dal using a pressure….”

 What’s ‘dal’ please, for us international readers? **We have added a simple definition of dal.**

 “There was fracture involving the right frontal sinus with pneumocephalus and 1×1 cm sized left frontal contusion (Figure 3).”

 The figure 3 referred to here is a brain, not a bone, window of the cranial CT. The frontal sinus fracture being described could therefore hardly be appreciated. We had actually wondered why the authors did not render some of the images at this frontobasal level in the bone windows. That’s what would have been more instructive for this part of the work. **We have added a new image of the bone window at the frontobasal level.**

# Reviewer report: Reviewer response for version 1

**DOI:** 10.5256/f1000research.17719.r40762

**Published:** 2018-12-03

**Authors:** Amit Agrawal

**Affiliations:** 1 Department of Neurosurgery, Narayana Medical College and Hospital, Nellore, Andhra Pradesh, India

In this article the authors report two interesting cases of head injuries. One is due to accidental explosion of a man-made device and another one is due to a natural calamity.

 In the second case, it is interesting to note that hailstorm is a natural phenomenon, and there are number of incidences reported in news ranging from minor injuries to fatal incidences both to humans as well as to livestock.

 As the authors have suggested, the systematic information for such injuries is lacking. This single case can be taken as message that we need to further expand the scope of the incidence and impact of injuries due to natural calamities on human life.

Are enough details provided of any physical examination and diagnostic tests, treatment given and outcomes?

Partly

Is the case presented with sufficient detail to be useful for other practitioners?

Partly

Is sufficient discussion included of the importance of the findings and their relevance to future understanding of disease processes, diagnosis or treatment?

Partly

Is the background of the case’s history and progression described in sufficient detail?

Partly

Reviewer Expertise:

NA

I confirm that I have read this submission and believe that I have an appropriate level of expertise to confirm that it is of an acceptable scientific standard.

# Response

**Published:** 2020-11-11

**Authors:** Joe M Das

**Affiliations:** College of Medical Sciences - Teaching Hospital, Nepal

In this article the authors report two interesting cases of head injuries. One is due to accidental explosion of a man-made device and another one is due to a natural calamity.

 In the second case, it is interesting to note that hailstorm is a natural phenomenon, and there are number of incidences reported in news ranging from minor injuries to fatal incidences both to humans as well as to livestock.

 As the authors have suggested, the systematic information for such injuries is lacking. This single case can be taken as message that we need to further expand the scope of the incidence and impact of injuries due to natural calamities on human life. **Thank you for understanding the major reason for including the second report in our article.**
